# Chapters on Diseases of the Ovaries, Translated from Kiwisch’s Clinical Lectures

**Published:** 1861-05

**Authors:** 


ART. III.—*Chapters on Diseases of the Ovaries, translated, by permission, from Kiwisch's Clinical Lectures on the Special Pathology and Treatment of the Diseases of Women; with Notes and an Appendix on the Operation of Ovariotomy.* By JOHN CLAY, M.R.C.S., Surgeon-Accoucheur to the Queen's Hospital, Birmingham, etc. etc. London: John Churchill, 1860. pp. 430.

THIS work, as we are informed in the preface, is a translation from Scanzoni's edition of the late Prof. Kiwisch's admirable work on the "Pathology and Treatment of the Diseases of Women, of the chapters relating to diseases of the Ovaries." Including certain notes and tables by Mr. Clay, and notes by Scanzoni, it purports to be a complete monograph on the subject of the ovaries, their diseases and treatment, and as such, we are led to anticipate a thorough handling of the subject in all its details. The editor gives some account of his efforts to obtain the materials for the work, and the difficulties encountered. He says:—

"I originally intended to have appended to Kiwisch's tables all the operations that were not included therein, down to the present time; but on comparing his collection with the cases as originally reported, it became obvious that the details as given by him were very scanty, and in many instances very inaccurate, and not available for practical purposes." * * *

While deliberating, under these circumstances, upon the best course to pursue, he was—

"Counseled to commence, *de novo*, by having recourse to the cases of ovariotomy as originally published, instead of trusting to the accuracy of compilers. This plan I at once proceeded to carry out, as far as was practicable; but as many American and some German cases were widely distributed over the periodical literature of each country, it was found impossible to procure them; and finding by comparison that the tables of Drs. Hamilton and Lyman, of America, and Dr. Simon, of Germany, were exceedingly accurate, such cases as I had not in my collection I have transcribed from these sources with adequate acknowledgment. * * * To insure accuracy, the tabulated details of each case operated upon in Great Britain, were forwarded to the respective operators for revision; reports of fresh cases were also solicited, and in many instances proofs were forwarded for correction. * * * *It is to be regretted that the details of many of the unsuccessful eases are not more ample.* * * *

"I have a less pleasing duty to perform, in justice to those who have so kindly aided me in my endeavors, in mentioning those who were not courteous enough to reply to my communications for particulars: these were Dr. Bird, London; Dr. Simpson, Edinburgh; and Mr. Terry, of Bradford. *It is a duty incumbent on all who have performed this operation to place the results of their experience before the profession, as by their undertaking it they have given some encouragement to others to follow their example.* With reference to Dr. Bird, these remarks are particularly applicable, as he has placed some of his perhaps more favorable experience before the profession, and, by the omission of full particulars of his more unsuccessful practice, has left his report in an exceedingly unsatisfactory state. It was on this account that I was particularly anxious to have correct details of diseases, and repeatedly forwarded papers to him; but I have not received even an acknowledgment from him. It is not for me to speculate on the causes which induced him to withhold the information, which has, as I have been informed, been often sought in vain by others as well as by myself."

We have been induced to give these lengthy extracts from the introduction of Dr. Clay, in order to show that such views obtain in other places as well as our own country. Too many men are fond of heralding their successes, but omit all notice of their failures, and from this cause it is that the profession are still unable to come to the proper conclusion concerning ovariotomy, in common with other remedial measures.

It was not until within the last century that any such means as ovariotomy was attempted for the relief of such cases as were afflicted with disease of the ovaries. Every plan that the wildest imagination could conceive of was employed to palliate, if not to relieve, the sufferings of these unhappy females. About the only method which seemed to offer any prospect of success was the tapping of the cyst when the contents were fluid, and thus diminishing its size.

The early history of the operation of ovariotomy was for a long time involved in much obscurity; the majority of writers, however, gave the credit of its first performance to L'Aumonier of Rouen, while others claimed it for Professor Galenzowski, or for Professor Dzondi. This subject having been carefully investigated by Professor Gross, then of Louisville, now of Philadelphia, he, in a paper read before the Kentucky

State Medical Society, in October, 1852, clearly demonstrated the incorrectness of these claims, and showed conclusively that Dr. Ephraim McDowell, of Danville, Kentucky, in 1809, performed the first recorded operation for the removal of the ovary. For a complete resumé of his cases, as well as a highly interesting biography of this distinguished surgeon, we refer our readers to the article, entitled "Origin of Ovariotomy: with an account of the Life and Services of the late Dr. Ephraim McDowell, of Kentucky," by Prof. S. D. Gross, published in the number of this journal for November, 1860.

The views of Professor Gross are now generally adopted, and at a meeting of the Obstetrical Society of London, held February 6th, 1861, Dr. Tyler Smith read a paper on *Ovariotomy*, in which he fully agrees in ascribing the full honor of its first performance to Dr. McDowell.* It is, therefore, with much surprise that we find not only that no allusion has been made to the originator of this operation, in the work before us, but the editor has carefully refrained from any further account of its history than the following: "It appears the operation was mentioned by *Schlenker* and *Williers* in the years 1723 and 1731. But it first came into favor in the present century principally; and within the last twenty years English and American physicians have interested themselves in its history," etc. Yet, as he places the case of L'Aumonier in his table of *successful cases of ovariotomy*, with the date of 1782, he thus deprives Dr. McDowell of his just rights as directly as though he openly denied the claim which has been made for the bold Western surgeon. This is a fault which is too common with our brethren "across the water;" whether from a jealousy of the rising giant of the western hemisphere, or a culpable negligence, we are unable to decide.

Since that time the operation has been performed very many times by operators in various countries; and in the tables furnished by Dr. Clay we have the records of 242 cases where one or both ovaries were removed, and the patients recovered from the operation; 183 cases where one or both ovaries were removed, but the patients died in consequence of the operation; 24 cases where the cyst was only partially excised, 14 of whom died; and 118 cases of *attempted* ovariotomy, where either no ovarian tumor existed, or the operation was abandoned; of the latter, 41 died.

But, from the tenor of Dr. Clay's prefatory remarks, as well as from our experience relative to the reports of physicians concerning their unsuccessful cases, we have ample room to believe that, were the list complete, the number of fatal cases would be vastly increased. Hence

* Med. Times and Gaz., London, Feb. 16, 1861.

arises the inquiry, "Is ovariotomy a justifiable operation?" This the editor (Dr. Clay) answers in the affirmative. He says, alluding to the tables above mentioned:—

"The tables show one fact, and which strikingly illustrates the advisability of the performance of the operation; and that is, out of 395 completed operations, 212 resulted in recovery. This is the more gratifying, as in many of the successful cases remedies were used previously to the operation being performed, and different operative procedures adopted with the hope of curing the disease or arresting its progress, but without success, and in many instances death was imminent. These cases of recovery may be regarded as triumphs of surgical skill, by means of which so many lives were secured, in several instances for years, which would otherwise have been lost to society. * * *

"From a careful review, therefore, of the whole of the facts connected with the operation of ovariotomy, as gathered from the work now translated, and from the preceding tables, I have no hesitation in expressing my opinion that *the operation is to be highly recommended in ovarian tumors under the circumstances previously narrated, as it is the only mode of removing a disease incurable by any other means.*"

Kiwisch, however, appears to be more in doubt upon the subject, regarding it as yet a matter of dispute, and only admissible under peculiar conditions, which he carefully lays down. He says:—

"At present it has been undoubtedly proved by many facts that women can lose an ovary, or even both by operation, without any considerable injurious consequences being caused thereby, (sterility in the latter case excepted.) The operation in some cases was also acceptable: for it freed the patient not only from a very painful, but also from a dangerous malady, and led to perfect health. As regards the question respecting the admissibility of the operation, which is next to be considered, the conditions must first of all be discovered under which such favorable results were obtained. * * * * *

"Even if all the cases of completed and attempted extirpations were known to us, no correct conclusion upon the advisability of the operation could be deduced from the results hitherto obtained, that is, from the numerical comparison alone; because the choice of the cases, the operative procedure, and the after-treatment produce essential changes in the results obtained, and therefore more favorable statistics may be reasonably expected in future, by a greater attention on the part of the operators to these indications. When we consider the results of the practice of some physicians, we must certainly be astonished at the successful results of the operations performed by them. Thus, Bird has operated 6 successive times with success; Clay, in 14 cases in which the operation was completed, cured 11 patients; Walne and Ephraim McDowell, in 6 extirpations lost only 1 patient; Lane, in 5 operations, had to lament 1 death only. On the other hand, several others have only unsuccessful cases to show. Thus, for instance, Lizars has operated four times: once he opened the body and found no tumor—the patient survived; a second time he was obliged to leave the operation uncompleted, in consequence of adhesions; a third time he found both ovaries diseased, and left one—the two latter patients recovered from the operation; while the fourth, in whom the operation was completed, died in fifty-six hours. Buhring also operated 4 times, and lost 3 patients, but saved the fourth, in whom the operation could not be completed by incision. We ourselves, also, have operated 5 times without success, and others have had similar results.

"It follows, from all that has been said, that, at present, it is very difficult to come to a well-grounded conclusion upon this operation; and we can, therefore, only endeavor in the following to approach the truth as nearly as possible. It is probable that about half of the patients hitherto operated upon have lost their lives, and generally in a few hours or days."

We can readily agree with Kiwisch, that this fact should be very seriously considered when an operation is proposed upon a person who is suffering but slight inconvenience by the malady, and where the life is not decidedly threatened.

Again, though the patient may not die immediately from the operation itself, yet in many instances the sequelæ are painful, tedious, and ofttimes terminate in death.

"Finally, it must yet be made the subject of inquiry in how far a relapse of the cancer did not take place in many patients from whom tumors, apparently cancerous, were removed, although there is no confirmatory experience known to us. It must be further mentioned that the operation, in some cases, is very difficult to perform, tedious, and painful, and must often be abandoned after it has been tolerably far advanced. Thus, very great adhesions, and uncommon breadth and shortness of the pedicle of the tumor, place insurmountable obstacles in the way of the operation; and although such incomplete operations carry off comparatively few individuals, at the moment, they make the condition of the patient much more deplorable than before."

After some remarks upon the difficulty of diagnosis, a subject upon which the profession of this city have recently been much enlightened by several valuable papers read by Dr. Washington L. Atlee before the Philadelphia County Medical Society, Professor Kiwisch remarks:—

"But, on the other hand, it must not remain unmentioned that the disease is often so distressing that the most urgent wish is expressed by the patient to be freed from the tumor, even with the apparent danger to life. In many cases, also, as the disease progresses, a longer continuance of life is not to be thought of, while in such cases the operation may have a perfectly satisfactory termination. Besides, this result was obtained in no small number of patients in a very short time, so that several could leave their bed in from three to five weeks. In some patients the operation was easily and quietly performed, and its immediate consequences were not always very distressing or unsatisfactory."

The importance of a correct diagnosis is especially dwelt upon, and it well may be, seeing that distinguished surgeons have laid open the abdomen and found no tumor; while others have fixed the day, assembled their assistants, and lo! the tumor had disappeared. To be sure, some of these errors can hardly be considered in any way excusable, as where the pregnant womb has been positively declared an ovarian tumor, or where a similar announcement has been made as to a collection of fæces.

It may be possible that some of us are too anxious to operate, to allow us carefully to examine the ground before hand. We feel a fear lest our case may in some mysterious way disappear, and leave us no opportunity of gaining distinction, and the applause of a wonder-stricken community.

There is not the least doubt existing in our mind, that as we become more and more skilled in the differential diagnosis of these tumors, the results will be far different from those hitherto obtained. The peculiar conditions under which ovariotomy would seem to be advisable are:—

"1. Continued progress of the disease which threatens danger to life, and yet leaves the hope that the operation will be borne. * * * * *

"2. In exceptional cases, small tumors even may urgently require the operation, when they cause symptoms which are dangerous; for instance, obstinate dropsy, symptoms of impaction, unmanageable ischuria, great tenderness, continued agrypnia, loss of appetite, etc., when these symptoms belong to a disease which permits the operation.

"3. Suitability of the case for operation. The free ovarian tumors, or those fixed by less extensive pathological adhesions, are most suited for the operation. Moderately firm adhesions may be separated successfully; while intimate adhesions of the tumor to the surrounding parts make the operation either impracticable, or the attempt has generally fatal consequences. * * *

"4. In simple, or compound cysts nearly allied to the simple, an attempt should previously be made with tapping in all cases.

"5. At the time when the extirpation is performed, the patient must be free from fever, and, if possible, afflicted with no other disease. In this respect, the internal sexual organs are to be examined with care, because it is frequently the case that ovarian diseases are combined with uterine."

It remains, then, for surgeons to diagnose carefully and positively, and for the patient to be laboring under positive danger to life, ere the operation, from the above views, would become justifiable. In addition, the fact must be taken into consideration that ovariotomy, a most formidable operation in its performance, is not the only means offered to the surgeon by which he may give comfort, and even in many cases, comparative health to his patient.

Under the head of *radical* treatment, Prof. Kiwisch gives us tapping; continually keeping open the puncture; injection into the open cyst; incision of the cyst; partial excision of the cyst. Many cases are recorded where, after tapping, the reaccumulation took place very gradually, or not at all. The method of tapping preferred by Kiwisch, as well as Scanzoni, is through the vagina, peritonitis not being liable to follow, as by the growth of the tumor the peritoneum is carried up before it.

The records of this operation are but few. Dr. Clay says :—

"It is surprising that an operation never hitherto immediately fatal, and so frequently successful, has not been more often practiced, especially conjoined with the injection of a solution of iodine, a mode of treatment which has been rarely attempted."

Scanzoni operated by this method 14 times: 8 cases were cured, in 2 the cysts refilled after a few weeks, 3 did not come again under his notice, and 1 died of typhoid fever two months subsequently.

Tapping through the abdominal walls is rather more dangerous, as the wounding of the peritoneum renders inflammation of that membrane extremely imminent. Another danger is the probable wounding of a large vessel, or, which not seldom occurs, the filling of the cyst with blood, and this internal hemorrhage causes great anæmia, even though it may not immediately jeopard life. The imperfect statistics of these operations will not enable us to do more than hazard a conclusion as to their permanently beneficial results, and we shall therefore pass to the consideration of the method of keeping open the cysts. Though success has resulted from this operation, yet too many objections to it exist to allow of its frequent performance. There is more liability to severe inflammation, and extensive destruction of tissue. Dr. Clay considers that "the success has not been such as to recommend it as an operation for general adoption."

Injections into the sac, especially of stimulating fluids, were at one time highly lauded, but the reports are very unfavorable. The inflammation thus set up may readily get beyond the power of the operator, and a rapidly fatal result ensue.

Incision into the cyst may often become necessary, when, on tapping, the contents of the sac are found to be of a viscid character. Where adhesions do not exist, the walls of the cyst may separate from those of the abdomen, and thus the contents of the tumor pass into the abdominal cavity. To prevent this, it is recommended to produce adhesions at the point of incision by the use of caustics, sutures, or any other means. Various operators have in a variety of ways performed this operation, and—

"From these trials it may be assumed that incision for the production of a radical cure is far more suitable than the first two methods we have mentioned, because it affords a perfect evacuation, and subsequently a proper discharge of pus and ichorous fluid, and consequently causes the necessary shriveling of the sac. But it must not be overlooked, that it is always an operation dangerous to life; and it may also be asserted with justice that, as regards its danger, it is not less than that of ovariotomy. In our opinion, therefore, the incision can only have a rational application in those cases in which extirpation is indicated, and which is impracticable in consequence of adhesions."

Lastly, tapping has been combined with internal medication. This has been divided into the constitutional, local, and after-treatment. The first consists of the use of mercurials till the gums are touched, diuretics and tonics, good diet, and exercise in the open air; the second, pressure to the tumor by bandages, thus checking the growth, and afterward, puncture and evacuation; the third, of continued application of a pressure bandage, and persistent medication.

This treatment, however, does not seem to have realized the anticipations of its proposers. "Still, in consequence of the comparatively slight danger attending it, it is worthy of further trial."

Some operators have suggested the excision of a part of the cyst, and the subsequent closure of the abdominal cavity. This seems to be an artificial imitation of the efforts of nature when spontaneous rupture of the cyst occurs. When the fluid of the tumor is not of an irritating nature, this may not produce any bad results, and a radical cure be produced.

The author deduces the following conclusions respecting—

"Internal and surgical treatment of simple ovarian cysts.

"1. There is no internal treatment known from which a radical cure of ovarian cysts may be expected with great probability.

"2. In the few cases in which a perfect cure resulted from internal remedies, the process of cure has remained unknown to us, and no conclusions, as to the treatment of other cases, have hitherto been deduced from them.

"3. The internal therapeutics are, therefore, chiefly symptomatic; and in those cases where a radical treatment is aimed at, it is always to be undertaken experimentally, and must not be carried so far that the patient suffers marked injury from it.

"4. The surgical treatment, apart from extirpation, is also divided into radical and symptomatic. For a radical cure, the vaginal puncture, according to the method taught by us, is particularly suited; and in cases where it is not practicable, the incision of the cyst from the abdominal coverings, particularly when extensive adhesions exist, and at any rate the last-mentioned excision of a part of the cyst wall, present the most proper treatment. But in all these methods of operation, it must not be overlooked that they are not without danger, and, consequently, are only to be performed in urgent cases. As a palliative treatment, abdominal tapping, both on account of its danger and the uncertainty of the relief it affords, should be performed only in dangerous or very distressing cases. In favorable, but at the same time very rare, instances, it may happen that, after its performance, either a perfect cure or a long-continued amelioration may be the result."

In concluding our review of this volume, we would express our satisfaction at the care and labor bestowed by Dr. Clay upon his portion, and especially the tabular arrangement of operations. These present, at a glance, an immense number of cases, with their history, treatment, and results, thus enabling the reader to draw his own inference as to the advisability of operations, without the necessity of groping amid a long and tangled array.

